# Akzidentelle Atemkalkingestion im Rahmen eines Tauchganges

**DOI:** 10.1007/s00101-021-00920-z

**Published:** 2021-02-16

**Authors:** Mark Michael, Noemi Freise, Verena Keitel, Andreas Schaper, Christian Plettenberg, Sven Dreyer, Michael Bernhard

**Affiliations:** 1 Zentrale Notaufnahme, Universitätsklinikum Düsseldorf, Moorenstraße 5, 40225 Düsseldorf, Deutschland; 2 Klinik für Gastroenterologie, Hepatologie und Infektiologie, Universitätsklinikum Düsseldorf, Düsseldorf, Deutschland; 3 Giftinformationszentrum-Nord, Universitätsmedizin Göttingen, Göttingen, Deutschland; 4 Klinik für Hals-Nasen-Ohrenheilkunde, Universitätsklinikum Düsseldorf, Düsseldorf, Deutschland; 5 Hyperbare Sauerstofftherapie, Universitätsklinikum Düsseldorf, Düsseldorf, Deutschland

**Keywords:** Atemkalk, Ingestion, Inhalationstrauma, Kolliquationsnekrose, Kreislauftauchgerät, Scrub, Ingestion, Inhalation trauma, Colliquation necrosis, Rebreather

## Abstract

Atemkalk wird in Kreislauftauchgeräten beim technischen Tauchen („Rebreathern“) verwendet. Vergleichbar mit einem Narkosekreislaufgerät dient bei einem halb- bzw. geschlossenen Atemsystem der Kalk der Kohlendioxidabsorbtion. Der Atemkalk enthält meist Kalziumhydroxid, das unter Wassereinwirkung zu Natronlauge reagieren kann. Bei der Ingestion bzw. Aspiration von Bestandteilen des Atemkalks kann es zu Verätzungen mit der Bildung von Kolliquationsnekrosen kommen. Eine frühzeitige Endo- bzw. ggf. Bronchoskopie ist hier zur Abschätzung von Folgeschäden essenziell.

## Abstract (en)

Breathing lime is used in closed circuit and semi-closed circuit rebreathers (CCR/SCR) for technical diving. Similar to the use in anesthesia systems, the lime usually contains calcium hydroxide, which can react to caustic soda under the influence of water. The ingestion of components of the soda lime can lead to burns of the esophageal mucosa with the formation of colliquation necrosis and the danger of esophageal perforation. Early endoscopy is essential in this case to assess the consequences of ingestion.

## Anamnese

Eine 57-jährige Patientin stellt sich in den Abendstunden an einem Werktag fußläufig in der zentralen Notaufnahme des Universitätsklinikums vor. Die Patientin ist eine erfahrene Taucherin (>350 Tauchgänge) und berichtet, am selben Tag erstmals einen Tauchgang mit einem Kreislauftauchgerät bzw. „semi-closed circuit rebreather“ [SCR] (Mares Horizon®, Fa. MARES S.p.A., Genua, Italien) unter Anleitung eines für dieses Gerät zertifizierten Tauchlehrers durchgeführt zu haben. Nach einer Tauchdauer von etwa 30 min habe die Patientin in 6 m Tiefe einen auffälligen Geschmack im Mundstück verspürt, woraufhin sie das Mundstück des Tauchpartners genutzt und dann ohne Notaufstieg aufgetaucht sei. Da bei einem SCR Atemkalk für die Elimination von Kohlenstoffdioxid (CO_2_) genutzt wird und ein dadurch verursachter „caustic cocktail“ erfahrenen Tech-Tauchern (anwesender Tauchlehrer) als Gefahr bekannt ist, habe die Patientin den Mund ausgespült und zeitnah Trinkwasser zu sich genommen. Initial seien Hustenreiz, Dyspnoe sowie eine Dysphagie aufgetreten. Daraufhin hatte sich die Patientin eigenständig mit ihrem Ehemann in einer Klinik ihres Heimatortes vorgestellt; von dort wurde sie an das rund 15 min entfernt liegende Universitätsklinikum verwiesen. Im Rahmen der Aufnahme über die zentrale Notaufnahme wurde die Patientin von den Notfallmedizinern auch Taucherärzten und Kollegen der Hals-Nasen-Ohren-Heilkunde vorgestellt.

## Initialbefund

Bei der Vorstellung in der zentralen Notaufnahme gibt die Patientin einen kurz nach dem Tauchgang aufgetretenen, seit etwa 2 h bestehenden und persistierenden Hustenreiz an. Zudem besteht eine ausgeprägte Dysphonie. Bei der Ersteinschätzung ergeben sich folgende Parameter: Blutdruck 145/86 mm Hg, Herzfrequenz 64/min, Atemfrequenz 18/min, pulsoxymetrische Sauerstoffsättigung (S_p_O_2_) 99 % unter Raumluft, Schmerzen 1/10 nach numerischer Rating-Skala (NRS). Nach der initialen Untersuchung werden pulsoxymetrisch Werte zwischen 90 und 96 % gemessen. In der klinischen Untersuchung ist eine diskrete Bronchospastik auskultierbar.

In der Gesamtschau (max. Tauchtiefe 6 m, Dauer 30 min, Nitrox 60, kein Notaufstieg) scheint ein Tauchunfall im Sinne einer Dekompressionserkrankung sehr unwahrscheinlich. Die Patientin erhält daher nur niedrig-dosiert Sauerstoff (4 l O_2_/min via Nasenbrille), worauf die S_p_O_2_ sich wieder normalisiert. In der körperlichen Untersuchung werden keine weiteren auffälligen Befunde erhoben. Der HNO-ärztliche Spiegelbefund zeigt enoral eine leicht gerötete Schleimhaut ohne Anhalt für Ulzerationen, flexibel-endoskopisch stellen sich Zungengrund, Epiglottis und aryepiglottische Falte schlank und ohne Zeichen eines Ödems regelrecht dar. Die Stimmlippen sind beidseits reizlos und regelrecht beweglich. Es bestehen keine Schwellungszeichen.

## Toxikologische Einschätzung

Durch ein vom Tauchpartner fotografiertes Behältnis kann die Art des Atemkalks eindeutig zugeordnet werden (Sofnolime 797 Grade I, Rebreather CCR Atemkalk), und es erfolgt die telefonische Rücksprache mit der Giftinformationszentrale. Mutmaßlich habe bei der Patientin durch einen unklaren Mechanismus eine Inhalation und/oder Ingestion mit diesem Atemkalk als „caustic cocktail“ stattgefunden. Nach den vorliegenden Daten der Giftinformationszentrale können die Bestandteile Kalziumhydroxid und Natronlauge (NaOH) eine ätzende Wirkung erzeugen, wobei die alkalische Reaktion im Vordergrund stehe. Bei Inhalation sind Hustenreiz, Hypoxie und die Entwicklung eines toxischen Lungenödems möglich. Eine symptomatische Therapie mit der Inhalation von Kochsalzlösung (NaCl, 0,9 %), Salbutamol/Ipratropiumbromid sowie die Gabe eines Antitussivums werden empfohlen und umgesetzt. Bei einer Ingestion ist die Möglichkeit einer Ösophagusverätzung gegeben, mit der potenziellen Entwicklung einer Kolliquationsnekrose und folgender Perforationsgefahr. Zusammenfassend wird eine stationäre Überwachung für 48 h empfohlen.

## Weiterer Verlauf

Die Patientin wird zur weiteren Therapie und Überwachung in die Notaufnahmestation der zentralen Notaufnahme aufgenommen. Unter symptomatischer Therapie, der Inhalation von Salbutamol, Ipratropiumbromid und 0,9 %iger NaCl-Lösung verspürt die Patientin eine subjektive Besserung; die S_p_O_2_ als objektiver Parameter normalisiert sich.

Eine Röntgenaufnahme des Thorax zeigt keinen wegweisenden pathologischen Befund, es zeigte sich eine diskrete Konturbildung rechts apikal am ehesten bei Überlagerung bzw. einer Hautfalte. Da ein ventraler Pneumothorax nicht ausgeschlossen werden konnte, wurde eine Verlaufskontrolle empfohlen.

Leichte Dysphagie, Dysphonie und Hustenreiz bestehen jedoch weiterhin. Zudem tritt im Verlauf ein stärkeres retrosternales Brennen auf, eine i.v.-Therapie mit Pantoprazol wird eingeleitet. Während initial eher eine pulmonale Symptomatik im Sinne eines Inhalationstraumas imponierte, wiesen die Dysphagie und das retrosternale Brennen auch auf eine Ingestion der Noxe hin.

Vor diesem Hintergrund erfolgt bei V. a. eine Laugeningestion die Rücksprache mit dem Endoskopiebereitschaftsdienst und bei aktuell diskreter Symptomatik, einer stabilen respiratorischen und hämodynamischen Situation wird bei führender gastrointestinaler Symptomatik eine Ösophagogastroskopie (ÖGD) am nächsten Morgen empfohlen und terminiert.

Differenzialdiagnostisch wird die Möglichkeit eines Tauchunfalls im Sinne einer Dekompressionskrankheit (DCS) in Betracht gezogen, hierbei ergeben sich aber weder in der Anamnese des Tauchgangs (Dauer 30 min mit Nitrox, max. Tiefe 6 m, kein Notaufstieg, kein Wiederholungstauchgang) noch in der körperlichen Untersuchung Hinweise wie z. B. eine ubiquitäre Schmerzsymptomatik, Pruritus [„Taucherflöhe“], Hautveränderungen oder ein fokal-neurologisches Defizit.

**Figure 1:**
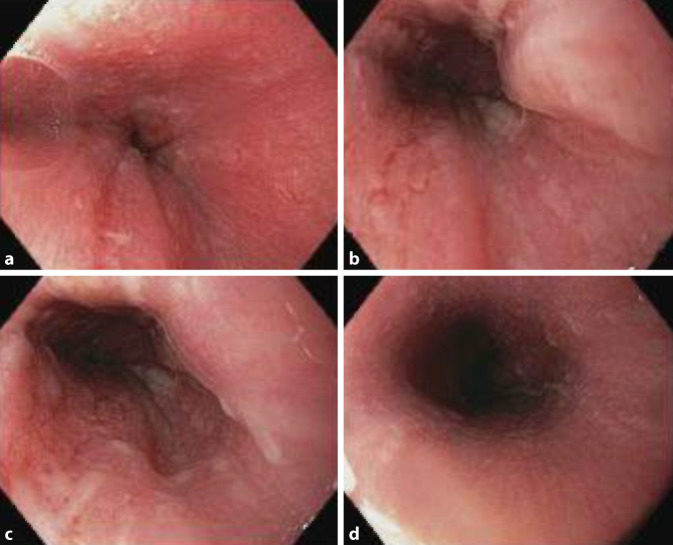

Die Patientin weist unter engmaschiger Überwachung am Folgetag eine regrediente Beschwerdesymptomatik auf. In der wie geplant durchgeführten ÖGD kann eine Verätzung des Ösophagus (Grad II, Erosionen und Erythem) nachgewiesen werden, es findet sich aber kein Hinweis auf eine Perforation. Stimmlippen und Larynx sind unauffällig ohne Hinweise auf eine Verätzung. Hämorrhagische flache Erosionen im Magen werden am ehesten einem initial stattgehabten Erbrechen zugeordnet (Abb. 1). Die Biopsien zeigen keinen Anhalt für eine Malignität; eine weitere endoskopische Therapie ist nicht erforderlich.

## Therapie und Verlauf

Zur weiteren Überwachung und Therapie wurde die Patientin auf eine Normalstation verlegt. Unter hochdosierter Einnahme eines Protonenpumpeninhibitors und regelmäßigen inhalativen Maßnahmen war eine rasche Befundbesserung zu verzeichnen; auch die initiale Dysphonie war im Verlauf rückläufig. Vier Tage nach dem Ereignis wurde die Patientin in gebessertem, stabilem Zustand in die häusliche Umgebung entlassen. Eine hochdosierte PPI-Therapie wurde für 4 Wochen empfohlen, zudem zunächst die Vermeidung harter bzw. unzerkauter Nahrung für eine bis 2 Wochen. Über eine sofortige Wiedervorstellung bei gastrointestinalen Blutungsstigmata, Schluckbeschwerden oder einer Verschlechterung des Allgemeinzustandes wurde die Patientin aufgeklärt. Vor weiteren Tauchgängen wurde die Vorstellung bei einem Taucherarzt zur erneuten Tauchtauglichkeitsuntersuchung empfohlen.

## Diskussion

Im vorliegenden Fall wird die Ingestion von Atemkalk im Rahmen eines Tauchgangs mit einem sog. Rebreather beschrieben. Bei unklarem Mechanismus der Ingestion (ggf. war die Patientin kurzfristig in Kopftieflage positioniert) bzw. ungeklärter technischer Ursache stand hier initial eine vermeintlich pulmonale Symptomatik im Vordergrund; im Verlauf manifestierte sich das klinische Bild einer Laugenverätzung des Ösophagus mit einem entsprechenden Korrelat im endoskopischen Befund.

Bei insgesamt milder Symptomatik konnte die Patientin nach stationärer Überwachung nach wenigen Tagen nach Hause entlassen werden. Aufgrund möglicher Folgeschäden nach dem Ereignis ist eine Verlaufskontrolle sinnvoll, und die Patientin wurde über mögliche Komplikationen aufgeklärt.

**Figure 2:**
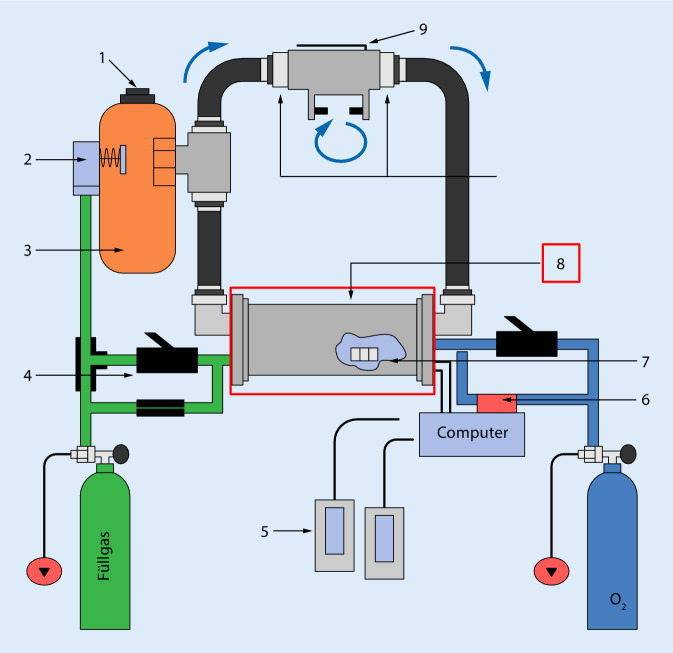

Der beschriebene Atemkalk wird in Form eines weißen, grobkörnigen Granulats (Größe etwa 1–2,5 mm) zur Kohlendioxid(CO_2_)-Absorption in Kreislaufgeräten eingesetzt (Abb. 2). Das Granulat besteht aus Kalziumhydroxid und NaOH. Bei der Bindung des CO_2_ wird auch mittels der Atemluft durch Wasser das CO_2_ zu Kohlensäure gebunden (H_2_CO_3_). Die Kohlensäure reagiert mit dem Natriumhydroxid des Atemkalks, einer Lauge, zu Natriumbikarbonat und Wasser. In einer weiteren Reaktion mit Kalziumhydroxid entstehen Kalziumcarbonat und Natriumhydroxid.

Pro 1000 g Atemkalk wird eine CO_2_-Bindungsfähigkeit von etwa 120 l beschrieben, dies ist allerdings temperaturabhängig, und die Wirksamkeit lässt bei niedrigeren Temperaturen nach.

Die Ingestion oder Inhalation von Atemkalk bei Tauchgängen ist in der Literatur nur vereinzelt beschrieben [1, 2]. Auch die Gesellschaft für Tauch- und Überdruckmedizin (GTÜM) listet dies nicht als gängigen Unfallmechanismus eines Tauchunfalls auf [3].

Insgesamt stellen Verätzungen des Ösophagus durch verschiedene Noxen eine seltene Entität dar. Rund 80 % der Noxeningestionen werden im Kindesalter beobachtet, hierbei ist meist ein akzidentelles Geschehen beschrieben, im Erwachsenenalter eher die Ingestion in suizidaler Absicht. Infrage kommende Substanzen sind alkalische Reinigungsmittel (Ofenreiniger, Abflussreiniger, Haushaltsreiniger) sowie saure Substanzen wie beispielsweise Batteriesäure, Zitronensäure, Essig oder Salzsäure. Das Ausmaß der Schädigung variiert dabei stark, und neben milden Verläufen sind auch kritische Fälle mit der Notwendigkeit intensivmedizinscher Maßnahmen und sogar einer chirurgischen Versorgung beschrieben [4].

Pathophysiologisch sind bei Verätzungen des Ösophagus die chemische Einwirkung von Säuren und alkalischen Lösungen zu unterscheiden. Säuren verursachen Koagulationsnekrosen mit konsekutiver Verschorfung. Alkalische Lösungen können Kolliquationsnekrosen bedingen, wobei es zu einer Penetration der Laugen in das tieferliegende Gewebe kommt und die Gefahr einer Organperforation besteht. Diese kann, abhängig vom Grad der Gewebeschädigung, über die Ausbildung von Ulzerationen nach Tagen zu Perforationen z. B. des Ösophagus führen, in schweren Fällen bereits Stunden nach der Ingestion. Im Falle einer auftretenden Mediastinitis ist diese mit einer hohen Letalität assoziiert.

Die Beschwerden der Patienten variieren mitunter stark und spiegeln nicht verlässlich das Ausmaß der Schädigung im Ösophagus oder im Magen wider. Etwa 20–60 % der Patienten zeigen neben der Verletzung des Ösophagus auch Schäden der Magenschleimhaut, die von einer einfachen Hyperämie und Erosionen bis zu ausgeprägten transmuralen Nekrosen reichen können. Eine akute Magenverletzung kann sich u. a. mit abdominellen Schmerzen oder Hämatemesis äußern.

Als Maßnahmen der Ersten Hilfe bei Säuren- und Laugeningestionen kann ggf. eine Verdünnung mit Wasser hilfreich sein. Vermieden werden sollten auf jeden Fall eine aktive Magenentleerung (z. B. induziertes Erbrechen) sowie die Gabe von Aktivkohle.

Bei Aufnahme des Patienten in der zentralen Notaufnahme stehen Basismaßnahmen wie die Labordiagnostik, symptomatische Maßnahmen (z. B. Inhalationstherapie), Volumengabe, die hochdosierte Therapie mit einem Protonenpumpeninhibitor sowie ggf. eine analgetische Therapie im Vordergrund. Eine Überwachung sollte in den ersten 24–48 h erfolgen. Bei septischem Verlauf oder hämodynamischer Instabilität sollte eine sofortige radiologische Diagnostik zum Ausschluss bzw. zum Nachweis einer Ösophagusperforation erfolgen. Eine enge Abstimmung mit der Gastroenterologie zur zeitnahen Endoskopie sollte erfolgen und eine Intervention rund um die Uhr verfügbar sein. Als Spätfolgen nach Ösophagusverätzungen können sich bei ausgeprägten Ulzerationen oder Nekrosen im Verlauf Strikturen bilden; zudem ist das Karzinomrisiko erhöht. Eine möglichst frühzeitige Endoskopie, nach Möglichkeit in den ersten 12–48 h, wird empfohlen. Die endoskopische Klassifikation ist wichtig zur Ermittlung des Schweregrades der Ösophagusverletzung (Tab. 1).

**Table 1:** 

Schweregrad	Charakteristika
*Grad 0*	Normale Schleimhaut
*Grad 1*	Oberflächliches Schleimhautödem und Erythem
*Grad 2*	Ulzerationen der Mukosa und Submukosa
Grad 2a	Oberflächliche Ulzerationen, Erosionen, Exsudationen
Grad 2b	Tiefe umschriebene oder zirkumferenzielle Ulzerationen
*Grad 3*	Transmurale Ulzerationen mit Nekrose
Grad 3a	Fokale Nekrose
Grad 3b	Ausgedehnte Nekrosen
*Grad 4*	Perforation

## Fazit für die Praxis

- Die Ingestion von Atemkalk bei einem Tauchgang stellt ein seltenes Ereignis dar, sollte aber differenzialdiagnostisch bei einem unklaren Tauchunfall berücksichtigt werden.
- Bei Verdacht auf eine Ösophagusverätzung ist eine zeitnahe Ösophagogastroduodenoskopie indiziert, um das Ausmaß der lokalen Schädigung abzuschätzen und eine Perforation frühzeitig zu erkennen.
- Als Akutkomplikation ist bei Verätzungen eine Ösophagusperforation beschrieben; Langzeitkomplikationen sind ebenfalls möglich.
- Bei schweren Verläufen ist ein strukturiertes, interdisziplinäres Management zu Atemwegssicherung, radiologischer Diagnostik, Endoskopie und ggf. chirurgischer Versorgung essenziell und muss daher 24/7 vorgehalten werden.
