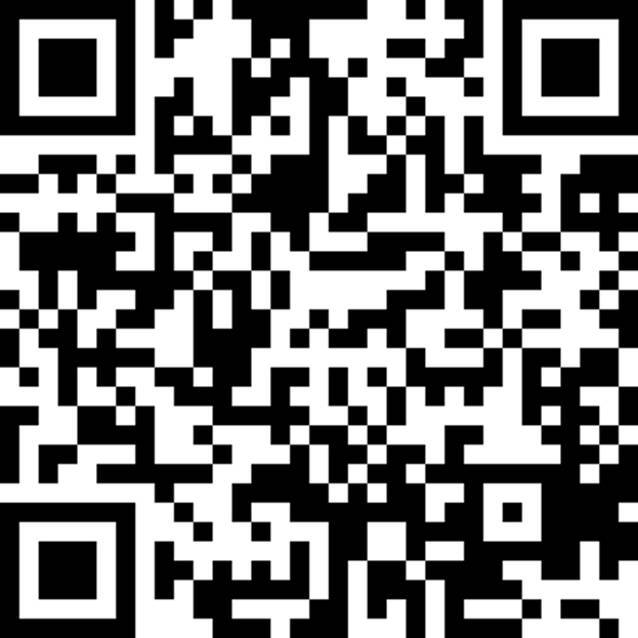# Erratum zu: Akzidentelle Atemkalkingestion im Rahmen eines Tauchganges

**DOI:** 10.1007/s00101-021-00981-0

**Published:** 2021-06-02

**Authors:** Mark Michael, Noemi Freise, Verena Keitel, Andreas Schaper, Christian Plettenberg, Sven Dreyer, Michael Bernhard

**Affiliations:** 1 Zentrale Notaufnahme, Universitätsklinikum Düsseldorf, Moorenstraße 5, 40225 Düsseldorf, Deutschland; 2 Klinik für Gastroenterologie, Hepatologie und Infektiologie, Universitätsklinikum Düsseldorf, Düsseldorf, Deutschland; 3 Giftinformationszentrum-Nord, Universitätsmedizin Göttingen, Göttingen, Deutschland; 4 Klinik für Hals-Nasen-Ohrenheilkunde, Universitätsklinikum Düsseldorf, Düsseldorf, Deutschland; 5 Hyperbare Sauerstofftherapie, Universitätsklinikum Düsseldorf, Düsseldorf, Deutschland

**Erratum zu:**

**Anaesthesist 2021**

10.1007/s00101-021-00920-z

In der Zusammenfassung des zuerst publizierten Artikels wurde die Zusammensetzung des Atemkalks nicht korrekt dargestellt. Bestandteile des Atemkalks sind Kalziumhydroxid und Natronlauge (NaOH). Hydroxycarbamid ist ein zytostatisch wirkendes Arzneimittel.

Der vollständige und korrigierten Artikel steht Ihnen auf www.springermedizin.de (Abb. 1) zur Verfügung. Bitte geben Sie dort den Beitragstitel in die Suche ein.

**Figure 1:**